# A missense substitution A49T in the steroid 5-alpha-reductase gene (*SRD5A2*) is not associated with prostate cancer in Finland

**DOI:** 10.1054/bjoc.2001.1789

**Published:** 2001-05

**Authors:** N Mononen, T Ikonen, K Syrjäkoski, M Matikainen, J Schleutker, T L J Tammela, P A Koivisto, O P Kallioniemi

**Affiliations:** Laboratory of Cancer Genetics, Dept. of Clinical Chemistry, Institution of Medical Technology, University of Tampere and Tampere University Hospital, P.O. Box 2000, FIN-33521 Tampere, Finland; Division of Urology, University of Tampere and Tampere University Hospital, P.O. Box 2000, FIN-33521 Tampere, Finland

**Keywords:** prostate cancer, 5-alpha-reductase, A49T, mutation

## Abstract

Prostatic steroid 5-alpha-reductase gene (*SRD5A2*) encodes a critical enzyme involved in the conversion of testosterone to dihydrotestosterone. A germline mis-sense substitution (A49T) leads to a variant SRD5A2 protein, which has a 5-fold higher in vitro V _max_ than the wild-type protein (Ross et al, 1998; Makridakis et al, 1999). The A49T variant was recently associated with 2.5 to 3.28-fold increased risk of prostate cancer (PC) in African-American and Hispanic men (Makridakis et al, 1999). Also, Jaffe et al (2000) reported an association between A49T and more aggressive disease among Caucasian patients. Here, we report that the prevalence of the A49T variant in 449 Finnish PC patients was 6.0%, not significantly different from 6.3% observed in 223 patients with benign prostatic hyperplasia or 5.8% in 588 population-based controls (odds ratio for PC 1.04, 95% C.I. 0.62–1.76 *P* = 0.89). There was no association between A49T and the family history of the patients nor with tumour stage or grade. Our results argue against a prominent role of the A49T variant as a genetic risk factor for prostate cancer development and progression in the Finnish population. © 2001 Cancer Research Campaign www.bjcancer.com


*British Journal of Cancer* (2001) **84**(10), 1344–1347
© 2001 Cancer Research Campaign
doi: 10.1054/ bjoc.2001.1789, available online at http://www.idealibrary.com on IDEAL®

http://www.bjcancer.com

### A missense substitution A49T in the steroid 5-alpha-reductase gene (*SRD5A2*) is not associated with prostate cancer in Finland

**N Mononen[1], T Ikonen[1], K Syrjäkoski[1], M Matikainen[1], J Schleutker[1], TLJ Tammela[2], PA Koivisto[1] and OP Kallioniemi[1]**

[1]Laboratory of Cancer Genetics, Dept. of Clinical Chemistry, Institution of Medical Technology, University of Tampere and Tampere University Hospital, P.O. Box 2000, FIN-33521 Tampere, Finland; [2]Division of Urology, University of Tampere and Tampere University Hospital, P.O. Box 2000, FIN-33521 Tampere, Finland

**Summary** Prostatic steroid 5-alpha-reductase gene (*SRD5A2*) encodes a critical enzyme involved in the conversion of testosterone to dihydrotestosterone. A germline mis-sense substitution (A49T) leads to a variant SRD5A2 protein, which has a 5-fold higher in vitro $V_{max}$ than the wild-type protein (Ross et al, 1998; Makridakis et al, 1999). The A49T variant was recently associated with 2.5 to 3.28-fold increased risk of prostate cancer (PC) in African-American and Hispanic men (Makridakis et al, 1999). Also, Jaffe et al (2000) reported an association between A49T and more aggressive disease among Caucasian patients. Here, we report that the prevalence of the A49T variant in 449 Finnish PC patients was 6.0%, not significantly different from 6.3% observed in 223 patients with benign prostatic hyperplasia or 5.8% in 588 population-based controls (odds ratio for PC 1.04, 95% C.I. 0.62–1.76, *P* = 0.89). There was no association between A49T and the family history of the patients nor with tumour stage or grade. Our results argue against a prominent role of the A49T variant as a genetic risk factor for prostate cancer development and progression in the Finnish population. © 2001 Cancer Research Campaign http://www.bjcancer.com

**Keywords**: prostate cancer; 5-alpha-reductase; A49T; mutation

Despite the substantial public health impact of prostate cancer, little is known about its aetiology. Improved knowledge of the natural history and genetic basis of prostate cancer would be important to develop prevention, screening, early diagnosis and intervention strategies. Recently, several loci have been implied in predisposing to prostate cancer by genetic linkage studies in cancer families. These include the HPC-1 locus at 1q24–q25 (Smith et al, 1996), HPC2 at 1q42–q43 (Berthon et al, 1998), HPCX at Xq27–q28 (Xu et al, 1998), HPBC at 1p36 (Gibbs et al, 1999), and loci at 20q13 (Berry et al, 2000), 11p (Gibbs et al, 2000) and 16q (Suarez et al, 2000). Exploration of the genetic basis of prostate cancer susceptibility by linkage analysis is challenging due to genetic heterogeneity (the large number of loci involved), due to incomplete penetrance of germline mutations and due to clustering of sporadic prostate cancer. Another approach to identify genetic risk factors is association, the study of the frequencies of polymorphisms in candidate genes between test and control populations. Several polymorphisms have been suggested to be associated with prostate cancer. These include the CAG and GGC repeats in the androgen receptor (*AR*) gene (Irvine et al, 1995; Giovannucci et al, 1997), as well as polymorphisms in the vitamin D receptor (Taylor et al, 1996; Ingles et al, 1997), 17-hydroxylase cytochrome P450 gene (*CYP17*) (Lunn et al, 1999), 3β-hydroxysteroid dehydrogenase type II (*HSD3B2*) (Devgan et al, 1997), BRCA1 (Ford et al, 1994) and BRCA2 (The Breast Cancer Linkage Consortium, 1999) as well as the 5α-reductase type II (*SRD5A2*) (Reichardt et al, 1995; Makridakis et al, 1997).

*Received 5 October 2000*
*Revised 22 February 2001*
*Accepted 28 February 2001*

*Correspondence to:* N Mononen

*SRD5A2* gene encodes for a critical enzyme catalysing the intra-prostatic conversion of testosterone, the most abundant androgen in the serum, to more potent 5α-dihydrotestosterone (DHT). DHT binds to the androgen receptor with high affinity and leads to transactivation of androgen targets. Since the development and growth of prostate cancer is dependent on androgens (Cunha et al, 1987), genetic variations that affect the *SRD5A2* enzyme activity could be associated with the risk of developing prostate cancer. A TA-dinucleotide repeat polymorphism at the 3′ UTR of *SRD5A2* (Davis and Russell, 1993; Reichardt et al, 1995; Kantoff et al, 1997) and a valine to leucine substitution at codon 89 (Makridakis et al, 1997; Febbo et al, 1999; Lunn et al, 1999) have not been found to be associated with prostate cancer. A third polymorphism, alanine to threonine substitution at codon 49 (A49T) results in a variant 5-α-reductase enzyme with a $V_{max}$ of about 5 times that of the wild enzyme (Ross et al, 1998; Makridakis et al, 1999). Makridakis et al (1999) recently reported that the A49T variant was more common among 216 African-American and 172 Hispanic prostate cancer patients than in the corresponding ethically matched controls. The authors suggested that up to 8% of the clinically advanced prostate cancers in these populations could be attributable to this genetic variant (Makridakis et al, 1999). This finding is supported by the findings of Jaffe et al (2000), who reported that men who carry the A49T variant have prostate tumours that are more likely to exhibit extracapsular extension and higher pTNM stage than men who do not carry this variant. In addition, the A49T variant was overrepresented in men with poor prognosis based on a combined analysis of their PSA level, Gleason score, and TNM stage, or on a combined analysis of their margin status and Gleason score (Jaffe et al, 2000).

Here, we compared the prevalence of A49T mutation among Finnish prostate cancer patients and population controls. The advantage of the Finnish population is its genetic homogeneity.

Therefore, ethnic and other population differences are likely to be less problematic than in case–control studies of more admixed populations.

### SUBJECTS AND METHODS

We collected genomic DNA specimens from 449 consecutively diagnosed prostate cancer patients and 223 benign prostate hyperplasia cases. These specimens came from the Tampere University Hospital, which serves as the local referral area for prostate cancer treatment. We also analysed 111 samples from 94 Finnish prostate cancer families from the whole Finland with at least 2 affected first-degree relatives. As controls, we acquired DNA specimens from 516 anonymous, unselected healthy male blood donors from Tampere region and 72 samples from autopsies in the Tampere University Hospital from males aged >65 years who were determined to be cancer-free. The study was based in a population-based sampling of prostate cancer patients and healthy blood donors and did not correspond to a classical case–control study. Taken together with the use of the Finnish population in this study, the typical problems associated with case–control studies should be avoided.

Written informed consent was obtained from all living patients and their family members, and research protocols were approved by the Ethical Committee of the Tampere University Hospital. The patients' family histories for malignancies were initially obtained from family questionnaires. All prostate cancer diagnoses were confirmed through medical records or from the Finnish Cancer Registry.

WHO tumour grading was available in 95% of the sporadic cases. Gleason score is not routinely used in Finland and was determined in 67% of the cases. The T-stage (T1–T4) was obtained in 92%, and M-stage in 81% of the cases. The M-stage was based on a bone scan in all evaluable cases.

Samples were first analysed by ASO-hybridization and later by minisequencing as this method was adapted in our laboratory. A large fraction of samples were analysed with both methods to ensure that the results were identical in all cases.

### Allele-specific oligonucleotide hybridization

Genomic DNA was amplified using primers 5′-ACTGGCCTTG-TACGTCGC-3′ and 5′-AGGGCAGTGCGCTGCACT-3′. Amplification reactions and conditions were as followed: 100 ng of DNA, 200 nM of both primers, 200 μM of each deoxy-NTP, 1.75 mM $MgCl_2$, and 1.5 U AmpliTaqGold™DNA Polymerase (Perkin-Elmer) in a final volume of 50 μl; at 95˚C for 10 min, followed by 35 cycles of 95˚C 1 min, 58˚C for 1 min, and 72˚C for 1 min, with a 5 min extension at 72˚C after the last cycle. Mutation detection was done using allele specific oligonucleotide (ASO) hybridization as described by Friedman et al (1995) with these exceptions: filters were prewetted in and wells washed with 0.4 M Tris-HCl pH 7.5, probes were end-labelled with $^{32}P$ at 37˚C for 3 h by Terminal Deoxynucleotidyl Transferase (Amersham Life Science), and hybridizations were performed at 54˚C. ASOs used in hybridizations were: 5′-GCCTGCCAGCCCGCGCCG-3′ (wild-type) and 5′-GCCTGCCAACCCGCGCCG-3′ (mutation). A mutation positive control as well as a negative control of the PCR reaction was included in each ASO hybridization.

### Minisequencing

A 174 bp fragment was first amplified as follows: 100 ng of DNA, 200 nM of both primers, 200 μM of each deoxy-NTP, 2.5 mM $MgCl_2$, and 2.5 U AmpliTaqGold™DNA Polymerase (Perkin-Elmer) in a final volume of 75 μl; at 95˚C for 10 min, followed by 35 cycles of 95˚C 1 min, 71˚C for 1 min, and 72˚C for 1 min, with a 5 min extension at 72˚C after the last cycle. Primers for PCR were 5′biotin-GCGAAGCCCTCCGGCTACGGGA-3′ and 5′-CGCGGGCACCCGCGAAGGAAGGC-3′. Minisequencing was performed as described by Syvänen (1998) with a detection primer 5′-CAGGAACCAGGCGGCGCGGG-3′.

Mutation positive ASO and minisequencing results were confirmed by sequencing with ABI PRISM 310 Genetic Analyser (Perkin-Elmer) as recommended by the manufacturer. Primers used in sequencing were the same as those in PCR.

### Statistical analysis

Statistical analyses were performed using the GraphPad InStat version 2.04a (GraphPad Software, CA, USA). Categorical variables were compared with the Fisher's exact test and $\chi^2$ (chi square) test for independence and continuous variables were analysed with Student's *t*-test. In addition, odds ratio (OR) and its 95% confidence intervals (95% CI) were calculated. All the tests were two-tailed.

### RESULTS

The frequency of the A49T substitution was 6.0% in 449 prostate cancer patients and 5.8% among the population-based controls (Table 1, OR = 1.04 for cancer, 95% CI = 0.62–1.76, $P = 0.89$). As compared to sporadic prostate cancer, the frequency of the A49T was slightly lower in 94 prostate cancer families (5.3%), whereas patients with benign prostatic hyperplasia had slightly higher frequency (6.3%). However, none of these differences were statistically significant. The prevalence of the A49T among the autopsy samples was 4.2% (3/72) and 6.0% (31/516) among the 516 blood donors. Because of the small number of the autopsy samples, we combined data for all donors as controls in the statistical analysis.

The results were also analysed separately for patients with metastatic (M1) and non-metastatic (M0) prostate cancer, as well as for patients with different T-stages, Gleason score and WHO grades (Table 2). No statistically significant associations emerged. In fact, there was a tendency for A49T substitution to be more common among the localized or lower grade cancers as defined either by a T1–T2 status, M0 status, Gleason score 2–6 or WHO grade I. However, none of these trends were statistically significant. The mean age of diagnosis did not differ between patients with A49T mutation (67.8 years, 27/449) compared to non-carrier patients (68.3 years, 422/449). The distribution for A49T genotype among population controls was in the Hardy–Weinberg equilibrium.

### DISCUSSION

Linkage studies published in prostate cancer have not shown any positivity at 2p23, the chromosomal location of the *SRD5A2* gene. However, Makridakis et al (1999) recently documented in a case–control association study that the A49T mutation in the *SRD5A2* gene may increase the risk of prostate cancer by a factor of 2.5 to

© 2001 Cancer Research Campaign 

**Table 1** *SRD5A2* genotype frequencies in 4 groups of Finnish patients and population controls

| | Prevalence of A49T (%) | Odds ratio[a] | 95% confidence interval | *P* value[a] |
|---|---|---|---|---|
| Sporadic prostate cancer | 27/449 (6.0) | 1.04 | 0.62–1.76 | 0.89 |
| Familial prostate cancer | 5/94 (5.3) | 0.92 | 0.35–2.40 | 1.00 |
| Benign prostatic hyperplasia | 14/223 (6.3) | 1.09 | 0.57–2.08 | 0.87 |
| Controls | 34/588 (5.8) | – | – | – |

[a]As compared to controls (blood donors and autopsy samples).

**Table 2** Association of *SRD5A2* genotype with clinicopathological characteristics of the prostate cancer patients

| | A/T or T/T | A/A | Prevalence of A49T (%) | Chi square | *P* value |
|---|---|---|---|---|---|
| T-stage | | | | 0.96 | 0.62 |
| T1–T2 | 14 | 200 | 6.5 | | |
| T2–T4 | 10 | 191 | 5.0 | | |
| Unknown | 3 | 31 | 8.8 | | |
| M-stage | | | | 0.94 | 0.62 |
| M0 | 20 | 274 | 6.8 | | |
| M1 | 3 | 65 | 4.4 | | |
| Unknown | 4 | 83 | 4.6 | | |
| Gleason score | | | | 1.83 | 0.40 |
| 2–6 | 15 | 182 | 7.6 | | |
| 7–10 | 4 | 99 | 3.9 | | |
| Unknown | 8 | 141 | 5.4 | | |
| WHO Grade | | | | 3.11 | 0.37 |
| I | 4 | 90 | 4.3 | | |
| II | 17 | 237 | 6.7 | | |
| III | 3 | 74 | 3.9 | | |
| Unknown | 3 | 21 | 12.5 | | |

3.28, in Hispanic and in the African-American populations. The risk for clinically advanced disease were shown to be 3.6-fold and 7.2-fold, respectively (Makridakis et al, 1999). The authors estimated that A49T could account for as much as 8% of clinically significant prostate cancers. Jaffe et al (2000) obtained a similar association for more aggressive disease among the Caucasian prostate cancer patients. In our study involving a total of 1371 subjects, the A49T *SRD5A2* mutation turned out to be equally common in population controls (5.8%), patients with benign prostatic hyperplasia (6.3%), and those with prostate cancer patients (6.0%). Stratification by T(N)M status, Gleason score or WHO grade did not reveal any subgroups with a significantly increased risk. Our results therefore do not support the findings by Makridakis and coworkers (1999) or those of Jaffe et al (2000). Finally, in our study the frequency of A49T was less common in index patients selected from 94 prostate cancer families as compared to 'sporadic' family-history-negative patients. If the A49T had a strong influence on the predisposition to prostate cancer, one would expect to find a higher frequency in the patients with positive family history. This finding further argues against the prominent role of A49T as a cause of prostate cancer in the Finnish population.

The differences between the studies may be explained by a number of factors. First, genetic risk factors may differ in different populations and can be influenced by environmental effects. Second, allele association studies are often affected by ethnic and other differences between the test and control groups that are unrelated to disease. Our study was based on materials from one university hospital region, which serves as the major regional referral centre for prostate cancer treatment in the area. In the Finnish health care system, patients in such major centres for cancer treatment reflect a representative unbiased sample of the entire population. The same is true for blood donors collected from the same hospital. The Finnish population, and particularly regions within the country, are genetically very homogeneous (Peltonen, 1997), and therefore ideal for allele association studies. Third, genetic associations often occur by chance. The number of prostate cancer cases analysed in our study was >2-fold higher than in either of the 2 populations studied by Makridakis et al (1999) or by Jaffe et al (2000). However, both study populations are still relatively small and larger studies are warranted. The main difference between our study and that of Makridakis et al (1999) is that the frequency of the AT/TT genotype in the Finnish control population (5.8%) was substantially higher than in the African-American (1.5%) and Hispanic controls (3.5%). The small group of samples from autopsy obtained from >65-year-old cancer-free Finnish men showed a 4.2% AT/TT genotype frequency. The results suggest that genotype frequencies in the control populations used in these studies varied more than those in the cancer patients. The AT/TT genotype was approximately equally common (range from 6.0% to 7.3%) among the prostate cancer patients in the different racial/ethnic groups in the 3 studies. The different results probably cannot be explained by differences in methods used to screen the A49T mutation.

Although sex hormones are clearly involved in the prostate carcinogenesis, the role of genetic variation in the genes comprising the steroid biogenesis pathway remains unclear. We

 *© 2001 Cancer Research Campaign*

conclude that the A49T variant of the *SRD5A2* gene is not strongly associated with prostate cancer development or progression in the Finnish population.

### ACKNOWLEDGEMENTS

We thank Minna Sjöblom and Kaisa Vaherto for excellent technical assistance. We also thank all the participating patients and families for their co-operation.

### REFERENCES

Berry R, Schroeder JJ, French AJ, McDonnell SK, Peterson BJ, Cunningham JM, Thibodeau SN and Schaid DJ (2000) Evidence for a prostate cancer-susceptibility locus on chromosome 20. *Am J Hum Genet* **67**: 82–91

Berthon P, Valeri A, Cohen-Akenine A, Drelon E, Paiss T, Wöhr G, Latil A, Millasseau P, Mellah I, Cohen N, Blanche H, Bellane-Chantelot C, Demenais F, Teillac P, Le Duc A, de Petriconi R, Hautmann R, Chumakov I, Bachner L, Maitland NJ, Lidereau R, Vogel W, Fournier G, Mangin P, Cohen D and Cussenot O (1998) Predisposing gene for early-onset prostate cancer, localized on chromosome 1q42.2–43. *Am J Hum Genet* **62**: 1416–1424

The Breast Cancer Linkage Consortium (1999) Cancer risks in BRCA2 mutation carriers. *J Natl Cancer Inst* **91**: 1310–1316

Cunha GR, Donjacour AA, Cooke PS, Mee S, Bigsby RM, Higgins SJ and Sugimura Y (1987) The endocrinology and developmental biology of the prostate. *Endocr Rev* **8**: 338–362

Davis DL and Russell DW (1993) Unusual length polymorphism in the human steroid 5 alpha-reductase type 2 gene (*SRD5A2*). *Hum Mol Genet* **2**: 820

Devgan SA, Henderson BE, Yu MC, Shi C-Y, Pike MC, Ross RK and Reichardt JKV (1997) Genetic variation of 3β-hydroxysteroid dehydrogenase type II in three racial/ethnic groups: implications for prostate cancer risk. *Prostate* **33**: 9–12

Febbo PG, Kantoff PW, Platz EA, Casey D, Batter S, Giovannucci E, Hennekens CH and Stampfer MJ (1999) The V89L Polymorphism in the 5α-reductase type 2 gene and risk of prostate cancer. *Cancer Res* **59**: 5878–5881

Ford D, Easton DF, Bishop DT, Narod SA and Goldgar DE (1994) Risks of cancer in BRCA1-mutation carriers. Breast Cancer Linkage Consortium. *Lancet* **343**: 692–695

Friedman LS, Szabo CI, Ostermeyer EA, Dowd P, Butler L, Park T, Lee MK, Goode EL, Rowell SE and King M-C (1995) Novel inherited mutations and variable expressivity of BRCA I alleles, including the founder mutation 185delAG in Ashkenazi Jewish families. *Am J Hum Genet* **57**: 1284–1297

Gibbs M, Stanford JL, McIndoe TA, Jarvik GP, Kolb S, Goode EL, Chakrabarti L, Schuster EF, Buckley VA, Miller EL, Brandzel S, Li S, Hood L and Ostrander EA (1999) Evidence for a rare prostate cancer-susceptibility locus at chromosome 1p36. *Am J Hum Genet* **64**: 776–787

Gibbs M, Stanford JL, Jarvik GP, Janer M, Badzioch M, Peters MA, Goode EL, Kolb S, Chakrabarti L, Shook M, Basom R, Ostrander EA and Hood LA (2000) Genomic scan of families with prostate cancer identifies multiple regions of interest. *Am J Human Genet* **67**: 100–109

Giovannucci E, Stampfer MJ, Krithivas K, Brown M, Brufsky A, Talcott J, Hennekens CH and Kantoff PW (1997) The CAG repeat within the androgen receptor gene and its relationship to prostate cancer. *Proc Natl Acad Sci USA* **94**: 3320–3323

Ingles SA, Ross RK, Yu MC, Irvine RA, La Pera G, Haile RW and Coetzee GA (1997) Association of prostate cancer risk with genetic polymorphisms in vitamin D receptor and androgen receptor. *J Natl Cancer Inst* **89**: 166–170

Irvine RA, Yu MC, Ross RK and Coetzee GA (1995) The CAG and GGC Microsatellites of the androgen receptor gene are in linkage disequilibrium in men with prostate cancer. *Cancer Res* **55**: 1937–1940

Jaffe JM, Malkowicz SB, Walker AH, MacBride S, Peschel R, Tomaszewski J, Van Arsdalen K, Wein AJ and Rebbeck TR (2000) Association of *SRD5A2* genotype and pathological characteristics of prostate tumors. *Cancer Res* **60**: 1626–1630

Kantoff PW, Febbo PG, Giovannucci E, Krithivas K, Dahl DM, Chang G, Hennekens CH, Brown M and Stampfer MJ (1997) A polymorphism of the 5 alpha-reductase gene and its association with prostate cancer: a case-control analysis. *Cancer Epidemiol Biomarkers Prev* **6**: 189–192

Lunn RM, Bell DA, Mohler JL and Taylor JA (1999) Prostate cancer risk and polymorphism in 17 hydroxylase (CYP17) and steroid reductase (SRD5A2). *Carcinogenesis* **20**: 1727–1731

Makridakis N, Ross RK, Pike MC, Chang L, Stanczyk FZ, Kolonel LN, Shi C-Y, Yu MC, Henderson BE and Reichardt JKV (1997) A prevalent missense substitution that modulates activity of prostatic steroid 5α-reductase. *Cancer Res* **57**: 1020–1022

Makridakis NM, Ross RK, Pike MC, Crocitto LE, Kolonel LN, Pearce CL, Henderson BE and Reichardt JKV (1999) Association of mis-sense substitution in SRD5A2 gene with prostate cancer in African-American and Hispanic men in Los Angeles, USA. *Lancet* **354**: 975–978

Peltonen L (1997) Molecular background of the Finnish disease heritage. *Ann Med* **6**: 553–556

Reichardt JKV, Makridakis N, Henderson BE, Yu MC, Pike MC and Ross RK (1995) Genetic variability of the human SRD5A2 gene: implications for prostate cancer risk. *Cancer Res* **55**: 3973–3975

Ross RK, Pike MC, Coetzee GA, Reichardt JKV, Yu MC, Feigelson H, Stanczyk FZ, Kolonel LN and Henderson BE (1998) Androgen metabolism and prostate cancer: establishing a model of genetic susceptibility. *Cancer Res* **58**: 4497–4504

Smith JR, Freije D, Carpten JD, Grönberg H, Xu J, Isaacs SD, Brownstein MJ, Bova GS, Guo H, Bujnovszky P, Nusskern DR, Damberg J-E, Bergh A, Emanuelsson M, Kallioniemi O-P, Walker- Daniels J, Bailey-Wilson JE, Beaty TH, Meyers DA, Walsh PC, Collins FS, Trent JM and Isaacs WB (1996) A genome wide search reveals a major suceptibility locus for prostate cancer on chromosome 1. *Science* **274**: 1371–1374

Suarez BK, Lin J, Burmester JK, Broman KW, Weber JL, Banerjee TK, Goddard KA, Witte JS, Elston RC and Catalona WJ (2000) A genome screen of multiplex sibships with prostate cancer. *Am J Hum Genet* **66**: 933–944

Syvänen AC (1998) Solid-phase minisequencing as a tool to detect DNA polymorphism. *Methods Mol Biol* **98**: 291–298

Taylor JA, Hirvonen A, Watson M, Pittman G, Mohler JL and Douglas AB (1996) Association of prostate cancer with vitamin D receptor gene polymorphism. *Cancer Res* **56**: 4108–4110

Xu J, Meyers D, Freije D, Isaacs S, Wiley K, Nusskern D, Ewing C, Wilkens E, Bujnovszky P, Bova GS, Walsh P, Isaacs W, Schleutker J, Matikainen M, Tammela T, Visakorpi T, Kallioniemi O-P, Berry R, Schaid D, French A, McDonnell S, Schroeder J, Blute M, Thibodeau S, Grönberg H, Emanuelsson M, Damber J-E, Bergh A, Jonsson B-A, Smith J, Bailey-Wilson J, Carpten J, Stephan D, Gillanders E, Amundson I, Kainu T, Freas-Lutz D, Baffoe-Bonnie A, Van Aucken A, Sood R, Collins F, Brownstein M and Trent J (1998) Evidence for a prostate cancer susceptibility locus on the X chromosome. *Nat Genet* **20**: 175–179

© 2001 Cancer Research Campaign
